# Research progress on postoperative higher-order aberrations after ICL implantation: patterns of change, influencing factors, and associated visual disturbances

**DOI:** 10.3389/fmed.2026.1764008

**Published:** 2026-03-13

**Authors:** Zonglong Hao, Liangliang Zhao, Ying Pei, Lili Nie

**Affiliations:** Department of Ophthalmology, The Second Hospital of Jilin University, Changchun, China

**Keywords:** higher-order aberrations, implantable collamer lens, refractive surgery, visual disturbances, visual quality

## Abstract

Implantation of the Implantable Collamer Lens (ICL) has become an effective surgical option for correcting high and extreme myopia, particularly in patients with insufficient corneal thickness or contraindications to corneal refractive surgery. Although numerous studies have demonstrated that ICL implantation can achieve favorable visual outcomes, some patients still experience postoperative visual disturbances, such as halos, starbursts, and ring-shaped visual disturbances, which are often accompanied by mild increases in higher-order aberrations (HOAs). This review summarizes the patterns and influencing factors of HOA changes following ICL implantation and explores the relationship between HOAs and postoperative visual disturbances, aiming to provide a theoretical basis for clinical practice and to offer a reference for improving postoperative visual quality.

## 1 Introduction

Myopia is the most common refractive error and one of the leading causes of visual impairment worldwide (1, 2). When myopia progresses to high myopia, it can lead to a range of complications, including retinal detachment (3). Currently, the global “myopia epidemic” is spreading, with an earlier age of onset, indicating that the prevalence of high myopia is likely to continue rising in the future (4, 5). Studies predict that by 2050, the global number of individuals with myopia will reach approximately 4.758 billion, approximately 938 million of whom will have high myopia (6).

Refractive surgery has become an important approach for correcting myopia. Current procedures include mainly corneal refractive surgery and intraocular refractive surgery. Patients with low to moderate myopia often prefer corneal refractive procedures, whereas those with high myopia are frequently ineligible because of limitations such as insufficient corneal thickness. In recent years, implantable collamer lens (ICL) implantation, a posterior chamber phakic intraocular lens procedure, has gradually emerged as a significant option for correcting high myopia owing to its reversibility, avoidance of corneal tissue removal, and wide range of refractive correction. ICL can correct myopia up to −18.0 D and astigmatism up to 6.0 D, providing a new surgical alternative for patients unsuitable for corneal refractive surgery (7). In 2014, the new ICL V4c lens was introduced into clinical practice. It features a central hole with a diameter of 0.36 mm in the optical center, which eliminates the need for peripheral iridotomy and facilitates aqueous humor circulation. This design effectively reduces the risk of cataract formation, minimizes corneal endothelial cell loss, and maintains normal intraocular pressure, thereby further enhancing the safety and clinical outcomes of ICL surgery (8–10).

Numerous studies have confirmed that ICL implantation demonstrates favorable safety, efficacy, predictability, and long-term stability in clinical practice (11–15). Notably, a study by Alkhabbaz et al. on the use of ICL in patients with keratoconus reported that 36% of eyes achieved an improvement of two or more lines in corrected visual acuity postoperatively, suggesting that ICL also has clinical value in correcting keratoconus-associated refractive errors (16).

With the development of refractive surgery, clinical demands have extended beyond simple visual acuity improvement, and the enhancement of postoperative visual quality has gradually become a focus for both clinicians and patients. Studies have shown that, compared with corneal refractive surgery, ICL implantation offers a more pronounced advantage in improving postoperative visual quality (17).

Higher-order aberrations (HOAs) are key factors affecting visual quality. Their importance in the evaluation of visual function has been confirmed by numerous studies, and they have become among the important quantitative indicators for clinically assessing visual quality (18–20). Similar to corneal refractive surgery, changes in HOAs after ICL implantation also significantly affect visual quality. In clinical practice, most patients who undergo ICL implantation are satisfied with their visual correction outcomes; however, some patients experience postoperative visual disturbances such as halos, glare, and starbursts. Related studies indicate that these symptoms are often accompanied by a concurrent increase in HOAs (21–25).

Although ICL implantation may induce changes in HOAs to some extent, the overall outcomes indicate that the procedure is highly effective for myopia correction and visual improvement and can significantly enhance the visual quality and the quality of life of patients. As one of the key factors affecting postoperative visual quality, the clinical significance of HOAs has not been fully recognized. On this basis, this systematic review of the patterns of HOA changes after ICL implantation, their influencing factors, and associated visual disturbances was conducted with the goal of offering clinicians a more comprehensive and in-depth theoretical reference and providing useful insights for future research and optimization in clinical practice.

## 2 Methods

### 2.1 Information sources and search strategy

PubMed and Web of Science (Core Collection) were searched from inception to February 20, 2026. Search terms combined ICL-related keywords (e.g., “implantable collamer lens,” ICL, “phakic intraocular lens,” V4, V4c, EVO, “central hole/port”) with HOA-related keywords (e.g., “higher-order aberration,” HOA, wavefront, aberrometry, coma, trefoil, spherical aberration). Full search strategies are provided in Supplementary Table 1.

### 2.2 Eligibility criteria

We included human clinical studies (prospective or retrospective) evaluating posterior chamber ICL implantation for myopia and/or myopic astigmatism, including ICL V4 and ICL V4c/EVO/EVO + models, with or without toric correction. Studies were eligible if they reported at least one HOA-related outcome (whole-eye/ocular, corneal, or internal; and/or coma, trefoil, spherical aberration) and provided extractable paired preoperative and postoperative HOA data at ≥ 1 postoperative time point. We excluded studies that were not posterior chamber ICL studies (e.g., corneal refractive surgery, cataract IOLs, anterior chamber phakic IOLs), did not report extractable paired HOA data, were non-clinical (*in vitro*/bench/model/animal), were ineligible publication types (reviews/editorials/letters/conference abstracts without full data), or were case reports/small case series ( < 10 eyes).

### 2.3 Study selection

Titles/abstracts were screened to remove clearly irrelevant records; records with insufficient information were retained for full-text assessment. Two investigators (ZLH and LLN) independently assessed full texts against predefined criteria, with disagreements resolved by discussion and adjudication by a third investigator (YP). The search identified 183 records (PubMed: 42; Web of Science: 141). After removing 36 duplicates, 147 records were screened and 109 were excluded. Thirty-eight full texts were assessed; 19 were excluded because paired pre- and postoperative HOA data were not reported or were not extractable. Nineteen studies were included in the qualitative synthesis (PRISMA flow diagram, Supplementary Figure 1).

### 2.4 Quality assessment

Methodological quality was assessed independently by two reviewers (ZLH and LLN). Comparative/cohort studies were evaluated using the Newcastle–Ottawa Scale (NOS), and case series were assessed using the Joanna Briggs Institute (JBI) critical appraisal checklist for case series. Disagreements were resolved by discussion and adjudication by a third investigator (YP) when necessary. Detailed results are provided in Supplementary Table 2.

### 2.5 Data extraction and synthesis

Two reviewers (ZLH and LLN) extracted data using a standardized form, including study design, sample size (eyes),follow-up time points, measurement device, measurement zone/pupil diameter, and HOA outcomes. Given substantial heterogeneity in devices, measurement zones/pupil diameters, follow-up schedules, and HOA components (whole-eye vs. corneal vs. internal), meta-analysis was not performed; evidence was synthesized qualitatively with structured stratification by measurement conditions and clinically relevant factors.

## 3. Basic concepts of HOAs

### 3.1 Fundamental theory of HOAs

The deviation between the actual wavefront produced by an optical system and the ideal wavefront generated by a perfect optical system is referred to as the wavefront aberration, which is a key metric for assessing the imaging quality of an optical system. Zernike polynomials are typically used to quantitatively represent wavefront aberrations. In clinical research, a 7th-order, 36-term Zernike polynomial is often employed for characterization. Terms of the 1st to 2nd order correspond to lower-order aberrations (LOAs), which can be corrected using spectacles, contact lenses, or refractive surgery to improve visual acuity. Terms of the 3rd order and above are collectively known as higher-order aberrations (HOAs), which cannot be corrected by conventional optical methods. Typical examples include spherical aberration, coma, and trefoil. Spherical aberration arises because peripheral rays of the pupil focus earlier than central rays and are closely associated with night vision quality, potentially causing halos around light sources, reducing contrast sensitivity, and impairing nocturnal vision. Coma manifests as a comet-like trailing effect in images, significantly affecting visual quality.

### 3.2 Common instruments for measuring HOAs

In ophthalmic clinical practice and research, a variety of instruments can be used to assess corneal, intraocular, and total-eye HOAs. Commonly used devices include iTrace (26), Pentacam HR (27), aberrometer, OPD-Scan III (7) and WASCA Analyzer (28). These devices can accurately quantify HOAs and assess overall visual optical performance, and they have been widely used for preoperative evaluation and postoperative follow-up in ICL implantation and other refractive surgeries.

## 4 Postoperative changes in HOAs

### 4.1 HOAs after ICL

Multiple studies have shown that HOAs change to varying degrees after ICL implantation, although the specific results differ among studies. Kayhan et al. compared changes in total eye, corneal, and intraocular HOAs after ICL implantation and reported significant differences in total and intraocular HOAs as well as coma, whereas corneal-related parameters did not significantly change, suggesting that ICL implantation has a limited effect on corneal morphology (29). Lin et al. reported that the early postoperative increase in HOAs was attributable mainly to corneal trefoil and intraocular spherical aberration, which may be related to the corneal incision and the optical characteristics of the ICL (7). Chen et al. reported a significant postoperative increase in total eye HOAs, coma, and trefoil aberrations (23). Niu et al. reported that postoperative coma and trefoil aberrations increased, whereas total HOAs and spherical aberrations did not significantly differ (30). Haiting et al. reported that both postoperative coma and spherical aberrations increased (31). Chen et al. demonstrated that HOAs exhibit dynamic changes: In the early postoperative period, intraocular and corneal trefoil aberrations increased significantly but returned to preoperative levels during later follow-up; intraocular spherical aberration slightly increased, possibly related to the optical properties of the ICL, whereas total eye spherical aberration remained stable, likely due to compensatory effects from refractive media such as aqueous humor and vitreous; moreover, total eye coma did not significantly change throughout the follow-up period (32). In summary, the changes in HOAs after ICL implantation are relatively complex, and differences among studies may be related to variations in measurement methods, follow-up duration, and individual patient factors. A summary of the characteristics of the enrolled studies is presented in Table 1. Tables 2–4 show pre- and postoperative HOAs of the whole eye, cornea, and intraocular structures measured at pupil diameters of 4, 5, and 6 mm, respectively, in different studies.

**TABLE 1:** Characteristics of the enrolled studies.

Authors	Year	Eyes (N)	Follow-up (months)	Measurement zone (mm)	Sphere (D)	Cylinder (D)	SE (D)	Measurement technique	Study design
Wei et al. (21)	2021	42	6	5	−8.14 ± 1.41	−0.24 ± 0.30	−8.26 ± 1.43	WASCA Wavefront Analyzer	Prospective study
Wei et al. (22)	2020	94	6	5	−7.60 ± 1.01	−0.94 ± 0.83	−8.07 ± 1.03	WASCA Wavefront Analyzer	Prospective study
Chen et al. (23)	2024	119	1	4	−8.67 ± 2.47	−1.34 ± 0.95	NR	OPD-Scan III	Prospective study
Wang et al. (27)	2024	122	3	4/6	−9.31 ± 2.43 − 8.85 ± 2.22	−1.34 ± 0.76 −0.82 ± 0.84	−9.98 ± 2.48 −9.26 ± 2.30	Pentacam HR	Retrospective study
Niu et al. (30)	2022	135	6	5	NR	NR	−10.16 ± 2.99	WASCA Wavefront Analyzer	Prospective study
Chen et al. (32)	2025	53	36	4	−8.09 ± 2.32	−1.25 ± 0.74	−8.72 ± 2.31	OPD-Scan III	Retrospective study
Aruma et al. (37)	2021	32	12	5	NR	−0.70 ± 0.40	−5.21 ± 0.73	WASCA Wavefront Analyzer	Retrospective study
Kamiya et al. (38)	2012	30	3	4/6	NR	1.11 ± 1.08	−4.79 ± 0.93	Hartmann-Shack Aberrometry	Retrospective study
Nassar et al. (48)	2023	40	3	NR	NR	−1.68 ± 0.82	−13.66 ± 2.23	Scheimpflug–Placido disc system, Sirius camera	Prospective study
Dan et al. (52)	2024	127	6	6.5	NR	NR	−8.54 ± 2.94 −7.98 ± 3.03	Pentacam HR	Retrospective study
Qin et al. (61)	2019	30	3	5	NR	NR	−13.87 ± 2.16	iTrace	Retrospective study
Shimizu et al. (72)	2012	58	3	4/6	NR	0.80 ± 0.55 0.78 ± 0.52	−7.52 ± 2.02 −7.57 ± 2.19	Hartmann-Shack Aberrometry	Prospective study
Wei et al. (76)	2022	36	6	4/6	−10.01 ± 1.47	−1.85 ± 0.75	−10.94 ± 1.41	iTrace	Retrospective study
Wan et al. (90)	2020	142	6	6	−4.74 ± 0.88 − 7.53 ± 0.78 −10.10 ± 1.01 −13.31 ± 1.54	−1.18 ± 1.06 −0.97 ± 0.64 −1.13 ± 1.04 −1.42 ± 0.97	−5.33 ± 0.83 −8.01 ± 0.82 −10.67 ± 0.94 −14.01 ± 1.47	HartmannOTERO_ WASCA aberrometer	Prospective study
Sinha et al. (117)	2022	25	6	4	NR	NR	−11.315 ± 2.131	iTrace	Prospective study
Li et al. (118)	2022	60	3	6	−5.67 ± 1.25	−0.74 ± 0.86	−6.05 ± 1.21	OPD-Scan III	Prospective study
Xu et al. (119)	2026	65	3	4	−7.43 ± 1.85 − 7.74 ± 1.93	−0.88 ± 0.23 −0.48 ± 0.31	−7.87 ± 1.87 −7.98 ± 1.92	Pentacam Axl	Prospective study
Li et al. (120)	2025	19	12	5	−7.33 ± 2.02	−0.67 ± 0.41	−7.66 ± 2.08	i Profiler Plus	Prospective study
Hosny et al. (121)	2013	20	1.4	NR	−12.90 ± 2.38	−1.15 ± 0.71	NR	Pentacam	Prospective study

SE, spherical equivalent; NR, not reported. Data are presented as mean ± standard deviation.

**TABLE 2:** Pre- and postoperative higher order aberrations of whole-eye, corneal, and intraocular with a 4-mm pupil.

Authors	Time	Whole-eye				Corneal				Intraocular			
		Total HOAs	Coma	Trefoil	Spherical aberration	Total HOAs	Coma	Trefoil	Spherical aberration	Total HOAs	Coma	Trefoil	Spherical aberration
Chen et al. (23)	Pre	0.16 ± 0.08	0.05 ± 0.03	0.12 ± 0.08	0.03 ± 0.02	NR	NR	NR	NR	NR	NR	NR	NR
	1mo	0.19 ± 0.08	0.07 ± 0.04	0.14 ± 0.08	0.03 ± 0.02	NR	NR	NR	NR	NR	NR	NR	NR
	*P*-value	0.01*	0.02*	0.01*	0.69	NR	NR	NR	NR	NR	NR	NR	NR
Chen et al. (32)	Pre	0.15 ± 0.07	0.06 ± 0.03	0.11 ± 0.08	0.03 ± 0.02	0.14 ± 0.05	0.08 ± 0.04	0.07 ± 0.05	0.05 ± 0.02	0.15 ± 0.05	0.07 ± 0.04	0.09 ± 0.05	0.04 ± 0.03
	1mo	0.21 ± 0.08	0.07 ± 0.05	0.17 ± 0.09	0.03 ± 0.02	0.17 ± 0.15	0.09 ± 0.07	0.11 ± 0.11	0.06 ± 0.02	0.21 ± 0.18	0.08 ± 0.07	0.13 ± 0.15	0.06 ± 0.03
	*P*-value	< 0.05*	> 0.05	< 0.05*	> 0.05	>0.05	> 0.05	< 0.05*	> 0.05	< 0.05*	>0.05	< 0.05*	<0.05*
Kamiya et al. (38)	Pre	0.10 ± 0.05	NR	NR	NR	NR	NR	NR	NR	NR	NR	NR	NR
	3mo	0.12 ± 0.06	NR	NR	NR	NR	NR	NR	NR	NR	NR	NR	NR
	*P*-value	NR	NR	NR	NR	NR	NR	NR	NR	NR	NR	NR	NR
Wei et al. (76)	Pre	NR	NR	NR	NR	NR	NR	NR	NR	0.172 ± 0.090	0.081 ± 0.044	0.086 ± 0.052	−0.038 ± 0.017
	6mo	NR	NR	NR	NR	NR	NR	NR	NR	0.102 ± 0.046	0.109 ± 0.096	0.056 ± 0.033	−0.088 ± 0.028
	*P*-value	NR	NR	NR	NR	NR	NR	NR	NR	<0.001**	0.155	0.008*	<0.001**
Sinha et al. (117)	Pre	0.371 (0.147−3.032)	0.211 (0.011−0.781)	0.212 (0.052−1.506)	0.007 (−0.136−1.310)	NR	NR	NR	NR	0.546 (0.101−3.021)	0.099 (0.034−1.001)	0.197 (0.054−1.548)	−0.029 (−1.01−0.693)
	6mo	0.185 (0.062−0.612)	0.067 (0.005−0.356)	0.121 (0.028−0.371)	−0.008 (−0.074−0.060)	NR	NR	NR	NR	0.168 (0.071−0.353)	0.049 (0.012−0.238)	0.080 (0.015−0.192)	−0.380 (−0.123−0.034)
	*P*-value	<0.0001**	0.0102*	0.0160*	0.0068*	NR	NR	NR	NR	0.0002**	0.0005**	0.0021*	0.4842

HOAs, higher order aberrations, NR, not reported. *p < 0.05, **p < 0.001. Data are presented as the mean ± standard deviation.

**TABLE 3:** Pre- and postoperative higher order aberrations of whole-eye, corneal, and intraocular with a 5-mm pupil.

Authors	Time	Whole-eye				Corneal				Intraocular			
		Total HOAs	Coma	Trefoil	Spherical aberration	Total HOAs	Coma	Trefoil	Spherical aberration	Total HOAs	Coma	Trefoil	Spherical aberration
Qin et al. (61)	Pre	0.485 ± 0.004	0.283 ± 0.003	NR	0.255 ± 0.003	NR	NR	NR	NR	NR	NR	NR	NR
	3mo	0.512 ± 0.002	0.286 ± 0.027	NR	0.258 ± 0.003	NR	NR	NR	NR	NR	NR	NR	NR
	*P*-value	<0.001*	0.105	NR	<0.001*	NR	NR	NR	NR	NR	NR	NR	NR
Wei et al. (21)	Pre	0.23 ± 0.08	0.15 ± 0.08	0.09 ± 0.06	0.06 ± 0.05	NR	NR	NR	NR	NR	NR	NR	NR
	6mo	0.27 ± 0.08	0.16 ± 0.08	0.16 ± 0.08	0.07 ± 0.05	NR	NR	NR	NR	NR	NR	NR	NR
	*P*-value	< 0.05*	> 0.05	< 0.05*	> 0.05	NR	NR	NR	NR	NR	NR	NR	NR
Wei et al. (22)	Pre	0.23 ± 0.08	0.13 ± 0.08	0.10 ± 0.06	0.06 ± 0.05	NR	NR	NR	NR	NR	NR	NR	NR
	6mo	0.26 ± 0.07	0.14 ± 0.08	0.15 ± 0.07	0.06 ± 0.05	NR	NR	NR	NR	NR	NR	NR	NR
	*P*-value	< 0.05*	> 0.05	< 0.05*	> 0.05	NR	NR	NR	NR	NR	NR	NR	NR
Niu et al. (30)	Pre	0.34 ± 0.88	0.12 ± 0.09	0.09 ± 0.08	0.06 ± 0.05	NR	NR	NR	NR	NR	NR	NR	NR
	6mo	0.28 ± 0.10	0.14 ± 0.09	0.16 ± 0.09	0.07 ± 0.05	NR	NR	NR	NR	NR	NR	NR	NR
	*P*-value	0.443	0.003*	0.000**	0.274	NR	NR	NR	NR	NR	NR	NR	NR
Aruma et al. (37)	Pre	0.26 ± 0.12	1.32 ± 1.00	1.03 ± 0.83	0.08 ± 0.06	NR	NR	NR	NR	NR	NR	NR	NR
	12mo	0.38 ± 0.18	1.40 ± 0.81	1.11 ± 0.87	0.08 ± 0.06	NR	NR	NR	NR	NR	NR	NR	NR
	*P*-value	0.004*	0.451	0.761	0.742	NR	NR	NR	NR	NR	NR	NR	NR
Li et al. (120)	Pre	0.24 ± 0.06	0.12 ± 0.05	0.12 ± 0.07	0.04 ± 0.07	NR	NR	NR	NR	NR	NR	NR	NR
	12mo	0.28 ± 0.10	0.13 ± 0.07	0.18 ± 0.10	0.03 ± 0.05	NR	NR	NR	NR	NR	NR	NR	NR
	*P*-value	0.129	0.682	0.038*	0.492	NR	NR	NR	NR	NR	NR	NR	NR

HOAs, higher order aberrations; NR, not reported,*p < 0.05, ** *p* < 0.001. Data are presented as the mean ± standard deviation.

**TABLE 4:** Pre- and postoperative higher order aberrations of whole-eye, corneal, and intraocular with a 6-mm pupil.

Authors	Time	Whole-eye				Corneal				Intraocular			
		Total HOAs	Coma	Trefoil	Spherical aberration	Total HOAs	Coma	Trefoil	Spherical aberration	Total HOAs	Coma	Trefoil	Spherical aberration
Kamiya et al. (38)	Pre	0.30 ± 0.12	NR	NR	NR	NR	NR	NR	NR	NR	NR	NR	NR
	3mo	0.41 ± 0.17	NR	NR	NR	NR	NR	NR	NR	NR	NR	NR	NR
	*P*-value	NR	NR	NR	NR	NR	NR	NR	NR	NR	NR	NR	NR
Wei et al. (76)	Pre	NR	NR	NR	NR	NR	NR	NR	NR	0.563 ± 0.232	0.269 ± 0.136	0.277 ± 0.121	-0.155 ± 0.074
	6mo	NR	NR	NR	NR	NR	NR	NR	NR	0.398 ± 0.101	0.301 ± 0.153	0.207 ± 0.110	-0.373 ± 0.053
	*P-*value	NR	NR	NR	NR	NR	NR	NR	NR	<0.001**	0.269	0.012	<0.001**

HOAs, higher order aberrations; NR, not reported, *p < 0.05, **p < 0.001. Data are presented as the mean ± standard deviation.

Notably, Zhao et al. emphasized that visual quality assessment should not focus solely on total HOAs but rather pay greater attention to the independent effects of specific aberration components. Researchers have suggested that different HOA components may collectively maintain an optical “balance” within the eye and that merely reducing a single aberration could lead to a decline in visual quality (33).

#### 4.1.1 Sources of heterogeneity and qualitative subgroup synthesis

Although all included studies evaluated HOA changes after posterior chamber ICL implantation, direct cross-study comparisons are constrained by substantial heterogeneity. To improve interpretability, we structured the synthesis around major sources of variation and performed a qualitative subgroup interpretation.

First, differences in measurement conditions represent a primary source of heterogeneity, particularly variability in pupil diameter/analysis zone (e.g., 4–6 mm or other zones). Qualitative stratification suggests that studies using larger analysis diameters are more likely to report greater magnitudes of coma/trefoil changes, whereas smaller zones more often yield attenuated or negligible changes. This pattern implies that pupil–effective optical zone interactions may partly explain discrepancies across reports.

Second, follow-up schedules influence the stability of measured outcomes. Early postoperative assessments (within weeks to approximately 1 month) are more susceptible to incision healing, transient inflammation, and tear-film instability, leading to greater variability, whereas later follow-up is more likely to reflect stable optical performance and longer-term lens positioning.

Third, non-uniform wavefront devices may affect absolute HOA estimates and the decomposition of aberration components. Accordingly, within-study pre-post comparisons and comparisons across studies with similar devices and measurement settings are more reliable than direct pooling across heterogeneous protocols.

Finally, ICL model/design factors (V4 versus V4c/EVO with a central port; toric versus non-toric) and lens-position metrics (tilt/decentration/rotation) were inconsistently reported, limiting rigorous subgroup comparisons. When positional parameters were available, larger deviations were more plausibly associated with increased coma-like aberrations, providing a mechanistic context for inter-study variability.

Overall, these qualitative subgroup considerations support cautious interpretation of between-study differences and underscore the need to standardize measurement conditions—particularly pupil/analysis zone, measurement device, and follow-up time points—to improve comparability and clinical translatability.

### 4.2 Comparison with other refractive correction methods

Theoretically, ICL implantation does not alter the physiological structure of the cornea, whereas corneal refractive surgery induces an increase in HOAs by reshaping the corneal morphology. Multiple studies comparing ICL implantation with corneal refractive procedures—such as Small Incision Lenticule Extraction (SMILE) and Laser-Assisted In Situ Keratomileusis (LASIK)—have generally shown that fewer HOAs are induced by ICL than by corneal refractive surgery (34–39). However, similar to studies on standalone ICL implantation, results differed among these comparative studies. Liu et al. reported that HOAs did not significantly change after ICL implantation but significantly increased following LASIK (40). Fu et al. reported an increasing trend in postoperative HOAs in both the ICL and SMILE groups (41).

In addition, some studies compared ICL implantation with other refractive correction methods. Compared with the simulated spectacle-corrected state, the ICL group showed a significant reduction in total eye and intraocular coma postoperatively, with greater improvement in coma observed in patients with greater preoperative refractive errors (42). Jiao et al. reported on HOAs following iris-fixated phakic intraocular lens implantation compared with ICL implantation and reported that HOAs were significantly greater in the former, with the difference in trefoil aberration being the most pronounced (43).

In terms of objective assessment, Qin et al. used the Optical Quality Analysis System II (OQAS II) and reported that visual quality was slightly better after ICL implantation than after SMILE in patients with high myopia (44). Moreover, OQAS II demonstrates good repeatability in evaluating retinal image quality (45). Other studies have further explored the influence of visual environments on postoperative visual quality. Zhu reported that under intermediate vision and low-light conditions, ICL implantation can induce a miosis effect, which reduces intraocular scattered light to some extent and thereby helps improve visual quality (46). Multiple studies have consistently demonstrated that compared with SMILE, ICL implantation provides superior postoperative visual quality (14, 26). In addition, a study by Kamiya et al. suggested that the optical quality of eyes after ICL implantation may not differ significantly from that of healthy control eyes (47).

From a temporal perspective, HOAs often tend to increase during the early period after ICL implantation, but they generally return to preoperative levels by three months post-operatively, with no statistically significant difference compared with baseline (48). In addition, some studies have indicated that although early postoperative changes in HOAs differ between the ICL and SMILE groups, no significant difference in total HOAs is observed between the two groups when the follow-up period is extended to 6 months or longer (49, 50).

Overall, existing studies consistently indicate that changes in HOAs after ICL implantation are complex and dynamic. These outcomes are influenced not only by measurement methods and follow-up duration but also by individual differences and the optical properties of the lens itself. Compared with corneal refractive surgery, ICL has a weaker impact on corneal biomechanics and morphology, induces lower levels of HOAs postoperatively, and demonstrates superior and more stable clinical advantages in improving visual quality.

## 5 Influencing factors of HOAs

### 5.1 Surgical incision

In ICL implantation, a corneal incision is required to insert the lens into the eye. This procedure itself may affect the morphology of the corneal surface and the intraocular optical system, thereby inducing corneal and intraocular aberrations. The presence of the corneal incision may also weaken the biomechanical integrity of the cornea, which in turn can influence postoperative HOA levels (51).

#### 5.1.1 Incision location

Multiple studies have confirmed that the location of the surgical incision is an important factor influencing postoperative corneal HOAs. Lin reported that the increase in corneal trefoil after ICL implantation is attributable mainly to the corneal incision (7). Dan et al. further compared the effects of clear corneal incisions and limbal tunnel incisions on postoperative HOAs. Although significant differences in posterior corneal coma and spherical aberration were observed between the two groups at 1 week post-operatively, both parameters returned to preoperative levels by 6 months, indicating that the two incision approaches have no significant long-term differences in their impact on HOAs (52).

Lever et al. first applied opposite clear corneal incisions (OCCIs) in cataract surgery to correct astigmatism (53). On the basis of this technical principle, Zhou et al. introduced the OCCIs technique into ICL implantation in an attempt to simultaneously correct astigmatism and evaluate its effect on corneal aberrations. Compared with conventional temporal incisions, OCCIs did not demonstrate an advantage in reducing HOAs and were associated with a significant increase in postoperative trefoil aberrations (54). A study by Song et al. also indicated that the location of the surgical incision is a key factor influencing changes in postoperative trefoil aberrations (55).

Wang et al. further investigated the role of incision location and morphology. In terms of incision location, superior incisions primarily induced corneal trefoil aberrations, whereas temporal incisions were more likely to affect corneal coma. In terms of incision morphology, incisions located farther from the corneal apex and with narrower widths were more favorable for reducing postoperative corneal HOAs, thereby improving postoperative visual quality (27). In addition, Li et al. reported that compared with scleral tunnel incisions, clear corneal incisions were more likely to induce additional corneal aberrations (56).

#### 5.1.2 Incision size

With respect to the impact of incision size on corneal HOAs, although studies specific to ICL are limited, the similarities between ICL and cataract surgery in terms of incision creation and surgical approach allow the abundant existing evidence from cataract surgery to serve as a useful reference. Tong et al. reported that patients who underwent small-incision cataract surgery (incision size of 3.0 mm) experienced significantly greater changes in total HOAs and corneal aberrations than those who underwent microincision cataract surgery (incision size of 1.5 mm) did (57). Similarly, Denoyer et al. compared microincision (1.7 mm) and small-incision (2.8 mm) cataract surgeries and reported that postoperative corneal HOAs were significantly lower in the microincision group than in the small-incision group (58). Kim et al. categorized patients on the basis of incision size into a small-incision group ( < 3.2 mm) and a large-incision group (3.2–4.5 mm). The results confirmed a significant correlation between incision size and trefoil aberration, with both groups showing a significant postoperative increase in spherical and trefoil aberrations. Notably, the large-incision group also exhibited a significant change in total HOAs (59). However, the findings of Yao differed, suggesting that compared with small-incision surgery, microincision cataract surgery did not significantly reduce corneal HOAs (60).

In addition, related studies have suggested that early postoperative changes in HOAs after ICL implantation are closely associated with surgical incisions. Qin et al. reported that spherical aberration increased significantly 1 week after ICL implantation but tended to decrease 3 months post-operatively. Researchers have speculated that these changes may be related to incomplete healing of the incision and a residual inflammatory response during the early postoperative period; as the incision gradually healed and inflammation subsided, the level of spherical aberration also decreased (61).

In summary, incision-related factors in ICL implantation—including incision location, size, and morphology—can influence postoperative HOAs to varying degrees, particularly during the early postoperative period. Although some effects gradually diminish or even return to preoperative levels as the incision heals and inflammation subsides, the evidence consistently highlights the important role of the surgical incision in determining postoperative visual quality after ICL implantation.

### 5.2 ICL-related factors

#### 5.2.1 ICL design

ICL lenses are designed with negative spherical aberration. As an intraocular implant, their advantage lies in not altering the corneal morphology, thereby preserving the biomechanical stability of the cornea (62). In contrast, SMILE surgery alters the original asphericity of the cornea, making it more flattened and thereby inducing a greater number of HOAs (22, 63, 64). In addition, some studies have suggested that ICL implantation may promote a more compact and symmetrical spatial arrangement of intraocular tissues, which helps reduce postoperative HOAs (42).

In ICL-related studies, postoperative changes in spherical aberration induced by the optical design of the ICL itself have received considerable attention. These studies include both *in vitro* and *in vivo* experiments, although their observations show some discrepancies. In terms of *in vitro* experiments, Vives et al. studied the ICL V4 model and reported that the lens optically exhibits negative spherical aberration, with the degree of negative spherical aberration increasing significantly with increasing refractive power (65).

Kim et al. conducted laboratory measurements using a Shack-Hartmann wavefront sensor and similarly confirmed that the ICL exhibits negative spherical aberration, which is positively correlated with its refractive power (59). In addition, Hashemian et al. reported that both ICL and TICL (Toric ICL) implantation can significantly induce negative spherical aberration, thereby partially compensating for the positive spherical aberration of the cornea (66). Similarly, in cataract surgery, selecting aspheric intraocular lenses can also introduce negative or zero spherical aberration into the ocular optical system to compensate for positive corneal spherical aberration (56). On the other hand, some studies have reported that in both the ICL and TICL groups, corneal and intraocular spherical aberrations increased significantly 1 month post-operatively (7).

Therefore, the negative spherical aberration design of the ICL has been shown, both theoretically and experimentally, to compensate for positive corneal spherical aberration, thereby contributing to the improvement in overall visual quality. However, current studies have not reached a consensus regarding the direction and magnitude of postoperative spherical aberration changes, which may be influenced by multiple factors, such as individual differences and lens refractive power, warranting further in-depth investigation.

#### 5.2.2 Presence or absence of a central hole

Whether the central hole design of the ICL V4c has an additional effect on postoperative HOAs and visual quality remains a subject of academic debate. Multiple studies have reported that, regardless of the presence of a central hole, postoperative visual quality is not significantly different, suggesting that the central hole design does not noticeably affect visual quality (67–70). Eppig et al. reported that although the cylindrical inner wall of the central hole may theoretically serve as a potential optical surface for inducing stray, its impact on-axis visual quality is not significant (71). Studies by Shimizu et al. also confirmed that, regardless of the presence of a central hole, the two types of ICL lenses showed no significant difference in postoperative changes in HOAs (72, 73). A clinical study by Huseynova further supported this view, indicating that the central hole design does not increase postoperative HOAs (74). However, some cohort studies have shown that although overall visual quality is similar after ICL V4 and ICL V4c implantation, the increase in HOAs is more pronounced in the ICL V4c group, particularly with a significant increase in spherical aberration (75).

#### 5.2.3 ICL vs. TICL

There is no significant difference between ICL and TICL implantation in terms of HOA induction (7). A study by Wei et al. similarly confirmed that the postoperative changes in HOAs induced in the two groups were not significantly different (21). Thus, in terms of postoperative HOA induction, ICL and TICL implantation yield relatively similar results, indicating that TICL does not induce more HOAs than ICL does.

#### 5.2.4 ICL position

**FIGURE 1:**
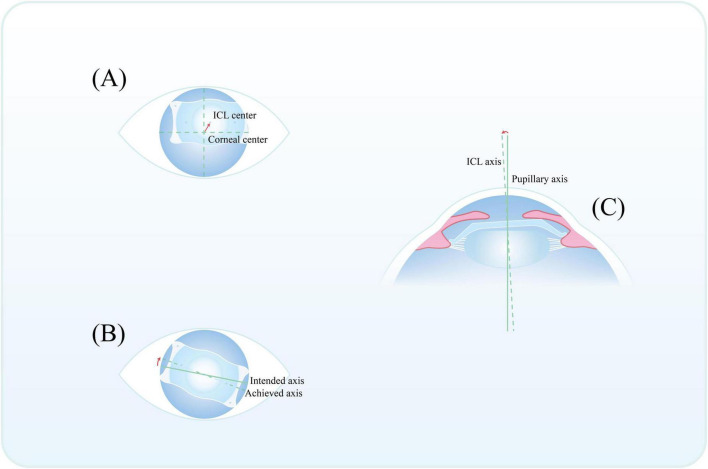
Schematic illustration of ICL positioning, including decentration **(A)**, rotation **(B)**, and tilt **(C)**. Decentration is calculated from the distance between the ICL center and corneal center **(A)**. Rotation is the angle between the intended axis and achieved axis of ICL **(B)**. Tilt is the angle between the pupillary axis and ICL axis **(C)**.

Tilt, decentration, and rotation can be quantified as the angle between the ICL optical axis and the pupillary axis, the distance from the ICL geometric center to the corneal apex, and the angular deviation between the actual ICL alignment axis and the intended correction axis, respectively (76, 77). As illustrated in Figure 1, the positioning parameters of the implanted ICL, including decentration, rotation, and tilt, can be evaluated using standardized schematic methods. Studies on intraocular lenses (IOLs) have fully confirmed that postoperative lens tilt, decentration, or rotation can significantly induce HOAs (78, 79), which in turn affect postoperative visual quality (80). On this basis, similar positional abnormalities after ICL implantation also carry the risk of inducing HOAs and impairing visual quality. Notably, the central hole of the ICL is often not aligned with the pupillary center (81); theoretically, this creates the potential for positional abnormalities such as ICL rotation and decentration (82). In addition, a study by Zhang et al. indicated that only approximately 21.6% of eyes had ICL haptics fully positioned within the ciliary sulcus after implantation (83).

The relationship between ICL positional abnormalities and postoperative HOAs remains controversial. Niu et al. reported that ICL tilt and decentration were not significantly correlated with postoperative HOAs; however, tilt was positively associated with the frequency, severity, and bothersome nature of nighttime visual disturbances (30). In contrast, Chen et al. suggested that mild ICL decentration may actually reduce trefoil aberration, as slight decentration allows the visual axis to pass entirely through the optical correction zone of the ICL, thereby avoiding diffraction effects from the edges of the central hole (23). Furthermore, other studies have indicated that when ICL decentration is less than 0.5 mm, its impact on intraocular HOAs is not significant (76). In addition, Pérez-Vives conducted an *in vitro* study and demonstrated that ICL decentration is positively correlated with coma, and this effect increases with increasing pupil diameter and increasing refractive power (84). Park et al. further standardized the ICL central hole decentration (using the 0.36 mm central hole diameter as the reference unit) and reported that when the central hole was displaced 1–2 standard units from the pupil center, total HOAs and trefoil aberrations were significantly greater than in eyes with a displacement of only 1 standard unit (85). In addition, some studies have reported that a higher postoperative ICL vault may cause upwards displacement of the central hole, but this does not affect visual quality (86).

With respect to tilt, some studies also did not observe a significant correlation with HOAs, likely because the tilt angle was too small to induce aberration changes that would affect visual quality (76). Park et al. reported that ICL rotation is related to the spherical power of the ICL (87). Wei et al. suggested that the vault is a potential risk factor for postoperative ICL rotation. ICL rotation is negatively correlated with vault and positively correlated with preoperative anterior chamber depth, indicating that precise ICL sizing can reduce the risk of rotation and other positional abnormalities (76). In addition, a clinical study on a novel ICL designed to correct both myopia and presbyopia revealed that patients who experienced a decline in visual acuity exhibited significantly increased coma, which researchers attributed to ICL positional abnormalities (88).

Overall, conclusions regarding ICL positional abnormalities—including tilt, decentration, and rotation—are inconsistent across studies; however, their potential impact on HOAs and visual quality should not be overlooked. Existing evidence suggests that mild positional abnormalities may have limited effects and, under certain conditions, may even provide some compensatory benefit. Nevertheless, when the degree of abnormality exceeds a threshold, it can significantly induce HOAs and impair visual quality.

### 5.3 Pupil

Multiple studies have confirmed that the pupil diameter is closely related to postoperative HOAs. When the pupil dilates, some light passes outside the effective optical zone of the ICL into the eye, generating diffraction effects and thereby inducing HOAs. The effective optical zone of the ICL is typically less than 6.0 mm and decreases with increasing refractive power, causing patients with high myopia to experience more pronounced increases in HOAs during pupil dilatation (76). In addition, Macedo-de-Araújo et al. (89) further suggested that strong light stimulation at night (e.g., during driving) can induce pupil dilation and physiologically trigger positive spherical aberration, thereby reducing visual quality. During the day, however, pupil constriction and compensatory effects of the crystalline lens reduce peripheral light entry and partially offset spherical aberration, thereby improving visual quality (89).

### 5.4 Other factors

Existing studies have shown that individual characteristics and preoperative refractive status can influence postoperative changes in HOAs after ICL implantation. Zhu et al. reported that the reduction in coma in patients after ICL implantation was positively correlated with spherical power, spherical equivalent, and ICL refractive power but negatively correlated with axial length. In the TICL group, lens thickness was also negatively correlated with a reduction in coma (42). Wan et al. grouped patients on the basis of the preoperative spherical equivalent (SE) and reported that compared with preoperative values, postoperative HOAs did not significantly differ across different refractive groups at 6 months (90). In addition, Zhu et al. demonstrated that at 3 months post-ICL implantation, patients with full preoperative spectacle correction and those with undercorrection showed significant differences in total and intraocular spherical aberration, with the undercorrection group tending toward a shift in spherical aberration toward negative values (91).

Chen et al. reported that the postoperative increase in intraocular spherical aberration was significantly correlated with patient age, preoperative corrected visual acuity, and postoperative residual astigmatism, whereas the increase in intraocular and corneal trefoil aberration at 1 month post-operatively was not significantly correlated with either preoperative or postoperative parameters (32). With respect to age, compared with younger patients, patients older than 40 years who underwent ICL implantation exhibited similar surgical safety, efficacy, and postoperative visual function (92). Further studies have confirmed that ICL V4c implantation also demonstrates long-term safety and efficacy in myopic patients over 40 years of age (93).

Therefore, individual characteristics such as refractive power, axial length, lens thickness, and age may influence postoperative HOA changes to varying degrees; however, their underlying mechanisms and clinical significance require further investigation. Table 5 shows the factors influencing HOAs following ICL implantation.

**TABLE 5:** Determinants of higher order aberrations following ICL.

Category	Determinants	Main findings	References
Surgical incision	Incision position	Corneal trefoil aberration is primarily induced by corneal incisions. No significant long-term difference in HOAs exists between clear corneal incisions and limbal tunnel incisions. OCCIs do not reduce HOAs and may increase trefoil aberrations; Superior incisions tend to induce trefoil, while temporal incisions more strongly affect coma. Incisions farther from the corneal apex and of narrower width are associated with fewer postoperative HOAs.	Lin et al. (7), Dan et al. (52), Zhou et al. (54), Song et al. (55), Wang et al. (27)
	Incision size	Larger incisions are more likely to increase postoperative HOAs in cataract surgery.	Tong et al. (57), Denoyer et al. (58), Kim et al. (59)
	Incision healing	Early postoperative HOA (esp. spherical aberration) increases due to incision healing and inflammation, then declines with wound recovery.	Qin (61)
ICL-related factors	ICL optical design	The ICL exhibits negative spherical aberration, which increases with lens dioptric power.	Vives et al. (65), Kim et al. (59), Hashemian et al. (66)
	Central hole (V4c)	There is no significant difference in postoperative HOAs or visual quality between ICLs with and without a central hole.	Eppig et al. (71), Shimizu et al. (72, 73), Huseynova et al. (74)
	ICL vs. TICL	There is no significant difference in postoperative HOAs induction between ICL and toric ICL.	Wei (21), Lin (7)
ICL position	Tilt	Mild tilt not significantly correlated with HOA.	Niu (30),Wei (76)
	Decentration	Mild decentration not significantly correlated with HOA. Slight decentration may even reduce trefoil. Excessive decentration increases coma and trefoil.	Wei et al. (76), Chen et al. (23), PérezVives et al. (84), Park et al. (85)
	Rotation	Rotation is associated with lens spherical power, anterior chamber depth, and vault.	Park et al. (87), Wei et al. (76)
Pupil	Pupil size	Larger pupils size is associated with higher HOAs and poorer visual quality.	Macedo-de-Araújo et al. (89), Wei et al. (76)

## 6 Common visual disturbances

**FIGURE 2:**
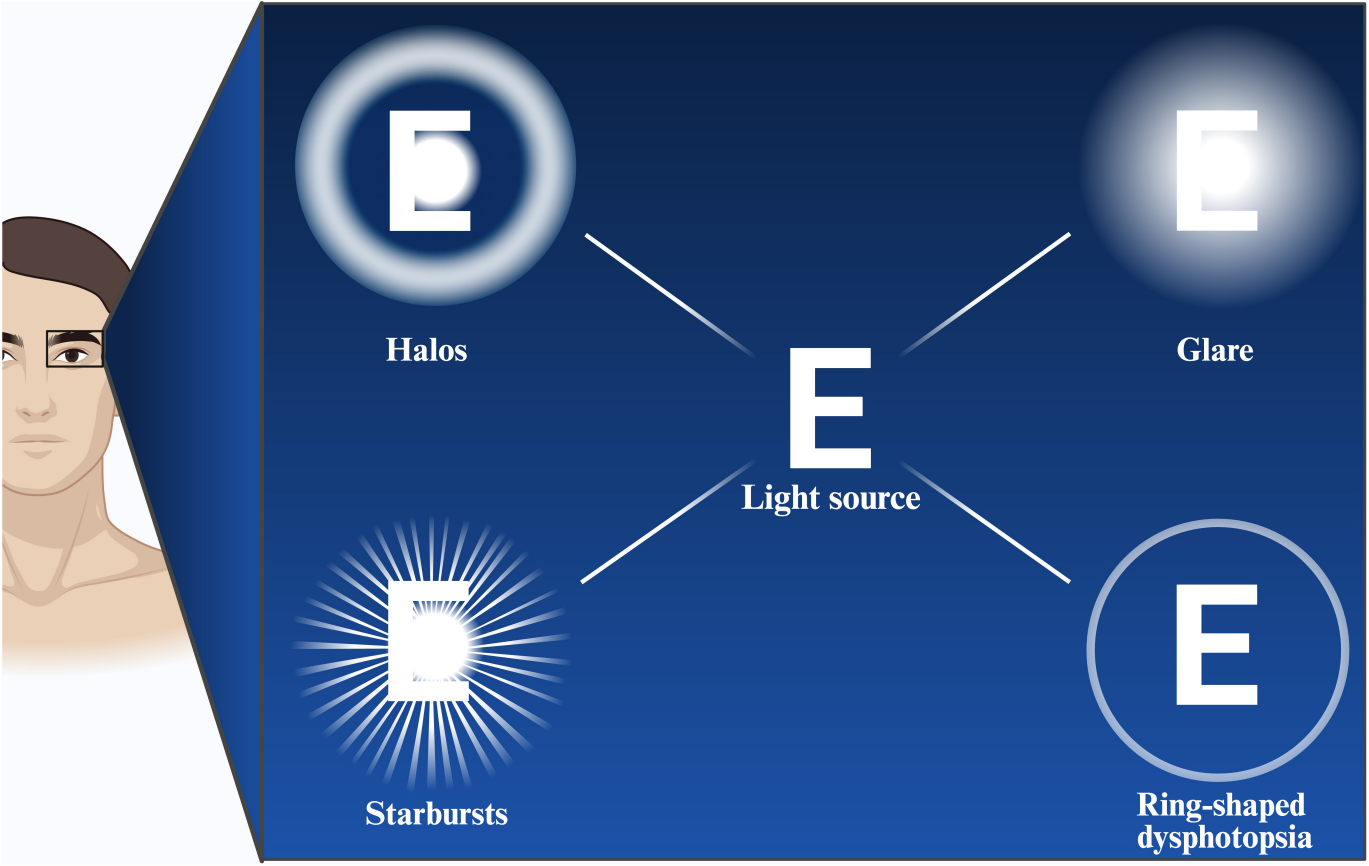
Schematic illustration of potential visual disturbances following ICL, including halos, glare, starbursts, and ring-shaped dysphotopsia. Created with BioRender.com.

Nighttime visual disturbances often occur in low-light environments, such as night driving or when looking at bright light sources, and are manifested primarily as halos, glare, and starbursts. As illustrated in Figure 2, potential visual disturbances following ICL implantation, including halos, glare, starbursts, and ring-shaped dysphotopsia, can be depicted schematically. With respect to the relationship between postoperative nighttime visual disturbances and HOAs after ICL implantation, the results of these studies have been inconsistent. Fu et al. suggested that although HOAs increase after ICL implantation, they are not significantly correlated with visual disturbances such as halos or starbursts (41). Studies by Aruma et al. (37) and Wei et al. (22) reached similar conclusions. Wei et al. suggested that this may be due to the generally mild nighttime visual disturbances experienced by patients and the relatively low levels of HOAs induced postoperatively (22). Similarly, Siedlecki et al. reported that after SMILE surgery, induced corneal HOAs were not clearly correlated with visual symptoms (94).

On the other hand, some studies suggest differences among various types of refractive surgery. Postoperative visual disturbances after LASIK are somewhat associated with HOAs, particularly starbursts, and the extent of starbursts is positively correlated with pupil diameter under low-light conditions (95).

Therefore, the relationship between postoperative HOAs and nighttime visual disturbances after ICL implantation remains inconclusive, likely because of the combined effects of surgical characteristics, individual variability, and physiological pupil changes.

### 6.1 Halos

Halos are the most common type of nighttime visual disturbance following ICL implantation. Puell proposed that when looking at a bright light source, forwards-scattered light within the eye forms veiling luminance on the retina, reducing retinal image contrast and thereby inducing halos (96). Previous studies have shown a high incidence of halos after ICL implantation; Wei et al. (22), Luo et al. (28), and Mohr et al. (97) reported incidence rates of 93.5, 91, and 90.1%, respectively. Although halos occur frequently, most patients report only mild discomfort (22, 97), and some studies suggest that this phenomenon can persist for more than 6 months (97).

Differences in halo manifestations have also been observed among various types of refractive surgery. Zhao et al. reported that mild halos were the most common visual disturbance after ICL implantation, whereas starbursts predominated after SMILE. Using the MonPack One visual testing device to measure halo size, they reported that in the SMILE group, the halo size increased significantly at 1 week postoperatively, decreased at 1 month, returned to baseline at 3 months, and stabilized at 6 months. In contrast, the halo size in the ICL group showed minimal overall change and remained stable (24). Fu et al. also reported that halos are the most common visual disturbance after ICL implantation, whereas starbursts are more prevalent after SMILE. They further reported that although postoperative HOAs increased significantly in both groups, there was no significant correlation between visual disturbances and HOAs (41).

Some studies have explored the potential mechanisms of halos. Lim et al. proposed that halo size is related to the difference between the pupil diameter and the ICL optical zone diameter, as well as the corneal horizontal diameter (98). Niu suggested that halos or glare are more likely to occur when a patient’s scotopic pupil diameter exceeds the maximum optical zone diameter (5.8 mm) of the ICL V4c (30). Other studies have also shown that the minimum pupil size during the light reflex is closely associated with halo size (99). Chen et al. further reported that patients with smaller pupils experienced milder postoperative halos and that ICL implantation could mitigate the adverse effect of preoperative refractive power on halo size (100). Zhu reported that patients with undercorrection preoperatively exhibited a greater incidence and greater severity of halos after surgery than fully corrected patients did and that halo severity was correlated with postoperative spherical aberration (91).

In a comparison of different ICL types, Wei et al. reported that the incidence of halos was significantly greater in the TICL group than in the ICL group and suggested that the degree of astigmatism corrected by TICL is a risk factor for halos and is correlated with the frequency, severity, and level of discomfort (21). With respect to the duration of postoperative halos, Liu et al. reported that 54.8% of patients experienced halos in the early postoperative period, which gradually subsided without any intervention (101). Similarly, Eom et al. reported that halos tended to diminish over the follow-up period, with an average duration of approximately 3 months (102).

In addition, Zhao et al. further investigated the relationship between halo size under glare-inducing conditions and HOAs. Their results revealed that at a luminance of 5 cd/m^2^, internal spherical aberration was negatively correlated with the halo radius, whereas the spherical equivalent had no significant effect. At 1 cd/m^2^ luminance, the mean pupil diameter, internal spherical aberration, and corneal HOAs substantially affected the halo radius (103)

### 6.2 Glare

Glare, another common nighttime visual disturbance after ICL implantation, differs from halos, which are described as glowing misty spheres surrounding a light source. Its mechanism is related mainly to contrast reduction caused by stray light from the light source (21). Qin, using the NEI-RQL-42 questionnaire, reported that compared with preoperative scores, glare scores decreased 3 months after ICL implantation. Researchers have suggested that the edges of the ICL central hole may induce glare to some extent, and this phenomenon could be related to patients’ early postoperative adaptation to the central hole (61). Approximately 65.2% of patients experience glare after ICL implantation, but among them, 63.0% considered the symptoms mild or not significantly bothersome (22). Wei et al. proposed that the severity of glare is closely related to scotopic pupil size (21). Ieong et al. reported that although ICL implantation can significantly improve overall quality of life, some patients still experience visual difficulties under glare conditions, such as nighttime driving (25).

### 6.3 Ring-shaped visual disturbances

In addition to halos and glare, another distinct type of nocturnal visual disturbance that may occur after ICL implantation is ring-shaped visual disturbance. From an optical perspective, Eppig suggested that the cylindrical inner wall of the ICL central hole may serve as an additional optical surface, refracting incident light and thereby producing a ring-shaped visual disturbance. This disturbance manifests as a relatively large and well-defined circular halo surrounding bright light sources (71). Eom et al. reported that 51.7% of patients experienced ring-shaped visual disturbances within 1 year after ICL implantation, with an average duration of 2.9 ± 3.8 months. The authors likewise attributed this phenomenon to the design of the ICL central hole (102). Martínez-Plaza et al. further confirmed that ring-shaped visual disturbances predominantly occur during the early postoperative period and become negligible by 6 months after surgery. Moreover, a study revealed that temporal decentration of the ICL was more likely to induce severe ring-shaped visual disturbances, whereas no significant correlation was observed with ICL power or preoperative pupil size (86, 104).

Although postoperative ICL implantation may be associated with increased HOAs and night vision symptoms such as halos and ring-shaped disturbances, these manifestations are generally mild and self-limiting. Given the advantages of ICL implantation in terms of refractive correction and the preservation of corneal biomechanical integrity, overall patient satisfaction remains high. Luo (28), Aruma (37), and Wei (22) reported postoperative satisfaction rates of 96.9, 93.8, and 96.1% among ICL patients, respectively, with 96.9, 90.6, and 88.2% of patients, respectively, indicating their willingness to recommend the procedure to others.

It should be noted that whether these nighttime visual disturbances persist in the long term remains controversial. Some studies have suggested that most symptoms gradually diminish and stabilize within 3–6 months post-operatively, indicating that the eye may require a certain period of adaptation to the implanted ICL (104, 105).

Although ICL implantation may lead to a certain increase in HOA, its correlation with nighttime visual disturbances (such as halos, glare, and annular visual disturbances) remains inconclusive. Most studies have shown a relatively high incidence of such symptoms, with halos being the most common. However, these disturbances are generally mild and self-limiting and typically diminish and stabilize within 3–6 months post-operatively. Their underlying mechanisms may be related to multiple factors, including ICL optical zone size, central hole design, pupil diameter, and positional abnormalities of the lens.

Overall, postoperative visual disturbances after ICL implantation do not appear to significantly compromise patients’ visual function or subjective satisfaction. Most studies have reported a postoperative satisfaction rate exceeding 90%, with a similarly high recommendation rate. These findings suggest that while certain nighttime visual disturbances may occur, ICL implantation effectively corrects high and very high myopia while preserving corneal biomechanical stability, thereby ensuring overall visual quality and quality of life.

## 7 Discussion

Clinical data indicate that most patients experience some degree of change in HOA after ICL implantation, although the patterns of change vary across different HOA types. Overall, the total ocular HOA, coma, and trefoil tend to increase slightly, whereas the spherical aberration generally decreases because of the negative spherical design of the ICL (23, 32). The underlying mechanisms may include the presence of corneal incisions, positional deviations of the ICL (e.g., decentration, tilt, and rotation), optical characteristics of the central hole, and early postoperative factors such as inflammation and tear film instability (27, 59, 61, 76). With postoperative healing and resolution of inflammation, HOA levels generally decrease and stabilize, with some patients even returning to preoperative levels (48). Notably, most comparative studies did not demonstrate a clinically meaningful additional induction of HOAs associated with toric correction or the presence of a central port (21, 72).

However, the literature on postoperative HOA after ICL implantation shows inconsistent results, which may be influenced by variations in preoperative refractive error, pupil diameter, measurement conditions, and devices among study populations, limiting the direct comparability of findings across studies.

From a clinical standpoint, the available evidence suggests that postoperative HOA changes and visual symptoms are not uniformly distributed across patients, but rather influenced by identifiable risk factors and modifiable perioperative variables. Larger mesopic/scotopic pupils, higher baseline corneal HOAs, and ocular surface instability may predispose certain subgroups to greater postoperative optical disturbances. At the same time, several factors remain clinically adjustable, including incision characteristics, lens sizing, and careful control of lens positioning. Organizing these considerations into a perioperative decision pathway may facilitate the translation of evidence into routine practice. A concise perioperative checklist is provided in Supplementary Box 1.

With respect to night-time visual disturbances, halos are the most common symptom following ICL implantation, with some patients also experiencing ring-shaped visual disturbances or glare (24, 102). However, most of these symptoms are mild and tend to diminish or disappear over time (104). Most studies have not established a significant correlation between postoperative HOA and symptoms such as halos, starbursts, or ring-shaped visual disturbances, suggesting that mechanisms beyond HOAs may contribute to symptom perception (22, 37).

Beyond HOAs, postoperative subjective visual disturbances after ICL implantation—such as glare, halos, and starbursts—are likely multifactorial and cannot be fully explained by HOA metrics alone. First, contrast sensitivity (CS) plays an important role in overall visual quality (26). Several comparative studies have shown that CS may improve or remain stable after ICL implantation (35, 38). These findings suggest that even when certain HOA components demonstrate mild changes, overall visual performance may still improve, thereby contributing to the apparent dissociation between HOA alterations and patient-reported symptoms.

Second, forward light scatter/straylight represents another critical determinant of night-vision quality (106). Recent studies evaluating postoperative optical quality after ICL implantation have incorporated objective metrics such as the modulation transfer function (MTF) cutoff and the Strehl 2D ratio (SR), which reflect retinal image quality, as well as the objective scatter index (OSI), which quantifies intraocular scatter (40, 85, 107). Importantly, halo size and severity have been reported to correlate independently with OSI, indicating that increased scatter may contribute to night-vision disturbances even when HOA changes are modest (108). In addition, ocular surface and tear-film stability may further influence scatter: postoperative tear-film instability can increase light scatter and degrade visual quality (109). Thereby weakening the direct association between measured HOAs and subjective visual perception.

Third, the relationship between pupil diameter and the effective optical zone of the implanted lens may amplify night-time symptoms. It is well established that ocular aberrations increase with pupil diameter (43). Moreover, prior studies have suggested that postoperative glare symptoms may be related to the mismatch between pupil size and the effective optical zone diameter of the implanted lens (110). Consequently, even when standardized HOA measurements show limited changes, patients may experience more pronounced visual discomfort.

Finally, visual perception is inherently subjective and encompasses multiple dimensions of image quality, including retinal image clarity, scatter, contrast sensitivity, and glare sensitivity. A single HOA-based metric is therefore unlikely to capture all aspects of patient-reported visual quality (111).

Given the current limitations in ICL postoperative HOA research—such as small sample sizes, short follow-up durations, and nonuniform measurement methods—future studies are needed. First, large-scale, multicenter, long-term longitudinal studies should be conducted to systematically monitor the dynamic changes in HOA after ICL implantation while simultaneously assessing the incidence, severity, and duration of night-time visual disturbances (e.g., halos, glare, and ring-shaped visual phenomena). It is also necessary to expand the range of myopic refractive errors to include mild ( ≤ −3.00 D), moderate (−3.00 to −6.00 D), high (−6.00 to −10.00 D), and ultrahigh ( > −10.00 D) myopia populations to comprehensively analyze HOA differences across refractive degrees. Second, standardized HOA measurement protocols should be established, with uniform pupil size settings, measurement environments, and instruments, to enhance the consistency and comparability of the results.

The correlation and underlying mechanisms between postoperative visual disturbances and HOA after ICL implantation remain unclear. Future investigations could integrate basic experimental studies with clinical research to elucidate the intrinsic relationship and regulatory mechanisms between the two and to develop targeted interventions aimed at reducing HOA levels and improving visual quality.

In addition, with the advancement of interdisciplinary medical engineering technologies, artificial intelligence has been applied to predict postoperative ICL vault and assist in preoperative ICL sizing, achieving promising results (112–114). On this basis, developing a predictive model for postoperative ICL HOA holds significant value. Such a model could integrate preoperative parameters—including corneal curvature, pupil diameter, axial length, lens thickness, and corneal topography—and utilize machine learning or deep learning algorithms to optimize the prediction of postoperative HOA outcomes. This approach would enable the design of individualized HOA correction strategies, thereby enhancing postoperative visual quality.

Finally, some studies have indicated that ICL implantation may transiently affect lacrimal gland function (115); dry eye has been shown to be associated with HOA, and interventions such as artificial tears can help improve HOA (116). Therefore, preoperative ocular surface assessment should be emphasized, and a comprehensive dry eye management protocol should be established, including routine screening, intervention, and follow-up, to maintain tear film stability and prevent its interference with HOA evaluation and visual quality.

## 8 Conclusion

The changes in HOAs after ICL implantation are complex and dynamic. Overall, in the early postoperative period, total ocular HOAs, coma, and trefoil tend to increase mildly, whereas spherical aberration may decrease because of the lens’ negative spherical design. HOA changes are influenced by multiple factors, including surgical incision characteristics (location, size, and shape), ICL-related factors (negative spherical design, the presence of a central hole, and toric correction), and lens positional abnormalities (tilt, decentration, and rotation). Although nighttime visual disturbances such as halos, glare, and ring-shaped phenomena may occur postoperatively, most are mild, self-limiting, and typically improve or disappear within 3–6 months. A thorough understanding of these findings can enhance insights into postoperative HOAs following ICL implantation, offering an important reference for the development of individualized intervention strategies and the optimization of postoperative visual quality.

## Author contribution

ZH: Writing – original draft. LZ: Writing – original draft. YP: Writing – review & editing. LN: Writing – review & editing.
