# Supplementary figures and images for: Research progress on postoperative higher-order aberrations after ICL implantation: patterns of change, influencing factors, and associated visual disturbances
Source: Front Med (Lausanne). 2026 Mar 13;13:1764008. doi: 10.3389/fmed.2026.1764008 (PMC13021442; doi:10.3389/fmed.2026.1764008)

Figure S1 PRISMA flow diagram of study selection.

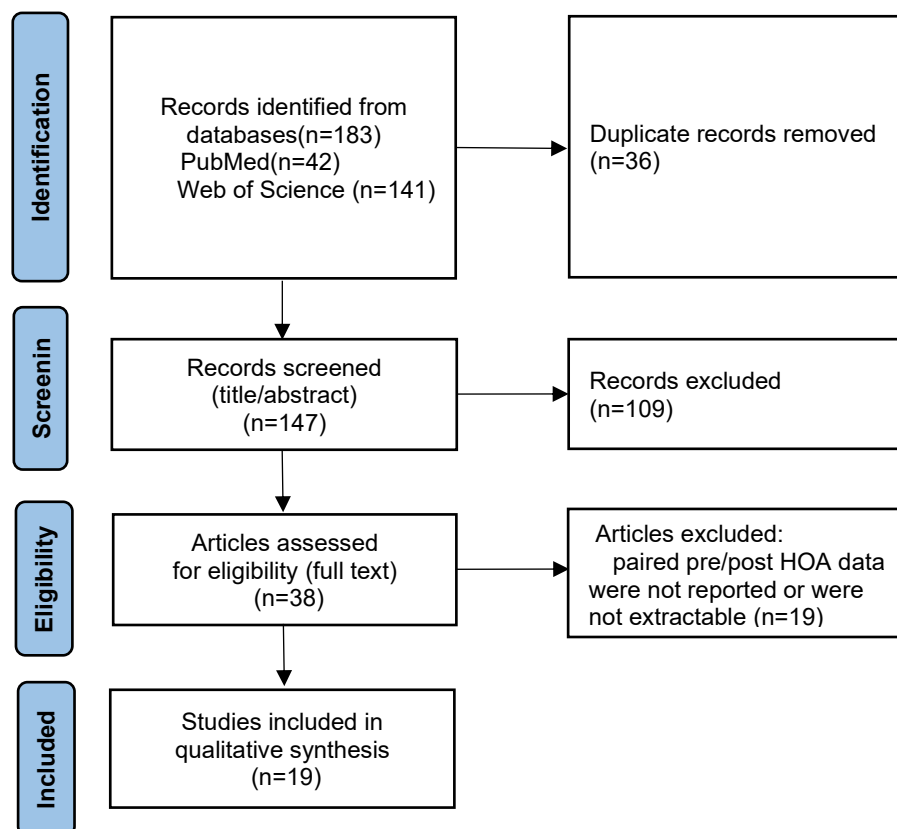

Supplement: Supplementary file 1 [file Image_1.pdf]
