# Supplementary material for: Research progress on postoperative higher-order aberrations after ICL implantation: patterns of change, influencing factors, and associated visual disturbances
Source: Front Med (Lausanne). 2026 Mar 13;13:1764008. doi: 10.3389/fmed.2026.1764008 (PMC13021442; doi:10.3389/fmed.2026.1764008)
Supplement: Supplementary file 2 [file Table_1.docx]

**Table S1 Full electronic search strategies.**

| **Database** | **Date searched** | **Time frame** | **Search strategy** | **Records retrieved** |
| --- | --- | --- | --- | --- |
| PubMed | 20 February 2026 | From inception to 20 February 2026 | (("implantable collamer lens"[Title/Abstract] OR ICL[Title/Abstract] OR "phakic intraocular lens"[Title/Abstract] OR "posterior chamber phakic"[Title/Abstract]) AND (V4[Title/Abstract] OR V4c[Title/Abstract] OR EVO[Title/Abstract] OR "central hole"[Title/Abstract] OR "central port"[Title/Abstract] OR "KS-aquaPORT"[Title/Abstract]) AND ("higher order aberration*"[Title/Abstract] OR "higher-order aberration*"[Title/Abstract] OR HOA[Title/Abstract] OR wavefront[Title/Abstract] OR aberrometr*[Title/Abstract] OR coma[Title/Abstract] OR trefoil[Title/Abstract] OR "spherical aberration"[Title/Abstract])) | n = 42 |
| Web of Science Core Collection | 20 February 2026 | From inception to 20 February 2026 | TS=(("implantable collamer lens" OR ICL OR "phakic intraocular lens" OR "posterior chamber phakic") AND (V4 OR V4c OR EVO OR "central hole" OR "central port" OR "KS-aquaPORT") AND ("higher order aberration*" OR "higher-order aberration*" OR HOA OR wavefront OR aberrometr* OR coma OR trefoil OR "spherical aberration")) | n = 141 |
