# Supplementary material for: Research progress on postoperative higher-order aberrations after ICL implantation: patterns of change, influencing factors, and associated visual disturbances
Source: Front Med (Lausanne). 2026 Mar 13;13:1764008. doi: 10.3389/fmed.2026.1764008 (PMC13021442; doi:10.3389/fmed.2026.1764008)
Supplement: Supplementary file 3 [file Table_2.docx]

Table S2 Quality assessment

| **Study** | **Design** | **Tool** | **Score** | **Overall risk** |
| --- | --- | --- | --- | --- |
| Aruma 2021 | Retrospective comparative  (EVO-ICL vs SMILE) | NOS | 7/9 | Low |
| Chen 2024 (AJO) | Prospective observational (decentration) | JBI | 8/10 | Low |
| Chen 2025 (BMC Ophthalmology) | Retrospective case series  (EVO-ICL) | JBI | 7/10 | Moderate |
| Dan 2024 (BMC Ophthalmology) | Retrospective comparative  (CCI vs limbus tunnel) | NOS | 6/9 | Moderate |
| Hosny 2013 | Comparative (ICL vs Cachet PIOL) | NOS | 6/9 | Moderate |
| Kamiya 2012 | Retrospective comparative  (ICL vs WFG-LASIK) | NOS | 7/9 | Low |
| Li 2022 (BMC Ophthalmology) | Prospective case-controlled  (ICL vs LASIK) | NOS | 7/9 | Low |
| Li2025 (Frontiers in Medicine) | Prospective contralateral eye comparative trial (PR vs EVO ICL) | NOS | 8/9 | Low |
| Nassar 2023 | Prospective interventional case series (ICL V4c) | JBI | 7/10 | Moderate |
| Niu 2022 (BMC Ophthalmology) | Observational study  (tilt/decentration effects) | JBI | 8/10 | Low |
| Qin  2019 (Medicine) | Observational case series  (EVO-ICL hypermyopia) | JBI | 7/10 | Moderate |
| Shimizu 2012 | Prospective intraindividual randomized comparison (Hole vs conventional ICL) | NOS | 8/9 | Low |
| Sinha 2022 | Prospective comparative  (ICL V4c vs RIL) | NOS | 7/9 | Low |
| Wan 2020 | Observational cohort with subgroup comparison (four myopia degrees) | NOS | 6/9 | Moderate |
| Wang2024(Frontiers in Medicine) | Retrospective comparative  (temporal vs superior CCI; morphology) | NOS | 6/9 | Moderate |
| Wei 2020 (Acta Ophthalmologica) | Prospective comparative  (ICL V4c vs SMILE) | NOS | 7/9 | Low |
| Wei 2021 (Acta Ophthalmologica) | Prospective comparative  (ICL vs TICL) | NOS | 7/9 | Low |
| Wei  2023 (Graefe’s) | Observational case series  (misalignment/rotation) | JBI | 7/10 | Moderate |
| Xu 2026  (BMC Ophthalmology) | Prospective non-randomized comparative (CRI vs conventional) | NOS | 6/9 | Moderate |
