# Supplementary material for: Research progress on postoperative higher-order aberrations after ICL implantation: patterns of change, influencing factors, and associated visual disturbances
Source: Front Med (Lausanne). 2026 Mar 13;13:1764008. doi: 10.3389/fmed.2026.1764008 (PMC13021442; doi:10.3389/fmed.2026.1764008)
Supplement: Supplementary file 4 [file Table_3.DOCX]

**Box 1 Perioperative Considerations for Visual Quality After ICL Implantation**

| **Preoperative: Risk Screening and Individualized Assessment** • Mesopic/scotopic pupil diameter (evaluate potential pupil–effective optical zone mismatch) • Baseline corneal HOAs • Ocular surface status (dry eye, tear-film instability, meibomian gland dysfunction) • Very high myopia and/or high night-vision demand (e.g., frequent night driving)  **Intraoperative: Modifiable Factors** • Careful incision planning (location and morphology) to minimize surgically induced corneal aberrations • Accurate ICL sizing • Meticulous implantation technique to reduce tilt, decentration, and rotation  **Postoperative: Follow-up and Reassessment Strategy** • Inform patients that early symptoms may improve with wound healing and neuroadaptation • Optimize ocular surface status before repeating wavefront measurements • For persistent or function-limiting symptoms, perform multimodal reassessment:  - HOAs (wavefront analysis)  - Objective optical quality/scatter metrics (e.g., MTF cutoff, Strehl ratio, OSI)  - Functional testing (contrast sensitivity, glare/halo quantification, defocus curves/functional depth of focus)  - Imaging-based evaluation of lens position (tilt, decentration, rotation) |
| --- |
